# Editorial: Next generation of omics analysis to study lipid-rich tissues

**DOI:** 10.3389/fendo.2025.1561447

**Published:** 2025-02-06

**Authors:** Isabella Piga, Tatjana Sajic

**Affiliations:** ^1^ Independent Researcher, Cagliari, Italy; ^2^ Faculty Unit of Toxicology, University Center of Legal Medicine, Faculty of Biology and Medicine, University of Lausanne, Lausanne, Switzerland; ^3^ Lausanne University Hospital and Geneva University Hospital, Lausanne, Switzerland

**Keywords:** proteomics, metabolomics, genomics, lipidomics, machine learning, blood plasma, human tissues

Advanced molecular analysis of “thousands” of human biological samples using genomics and transcriptomics technology is accelerating basic research along with mass spectrometry-based omics phenotyping, enabling detailed proteomics, metabolomics, glycomic, and metallomics characterization. These omics datasets, combined with demographic information (geographic location, age, gender, race, ethnicity, socioeconomic status), including daily biometric data (smartwatches, lifestyle, diet), are contributing to the generation of vast data resources available to researchers (1–3)for the molecular understanding of various human health issues (4–8). The rapid development of artificial intelligence (AI) technologies and their application to massive data resources promises to revolutionize medical practice and improve the therapeutic and diagnostic management of various human pathologies (2, 6, 9).

Hand in hand with these advances, a high-quality omics dataset of biological samples that can be linked to patient medical records is a prerequisite for the collection of valuable quantitative data resources using sensitive instrumentation.

Mammalian tissues have a complex chemical composition and contain molecules with different physical and chemical properties (e.g. lipids, proteins) that interact with each other and affect their omics phenotyping. For example, specialized lipid-rich tissues with heterogeneous molecular composition and high molecular dynamic range, such as blood plasma, adipose tissue and brain tissue, can be challenging for the analysis of molecules of interest (proteins, metabolites, DNA or RNA) and their mutual interactions. To compensate for confounding factors in the analysis, as well as analytical measurement errors, high sample-to-sample variability, missing measurements, batch effects, or feature selection error rates, novel software tools and statistical approaches are being developed to help researchers manipulate data and extract meaningful biological information.

This Research Topic includes four original articles that provide an in-depth exploration of the circulating blood lipidome and its alterations in various pathological conditions. The studies cover a range of clinical contexts, from metabolic disturbances, such as pre-metabolic and metabolic syndromes, to neurodegenerative diseases, particularly focusing on Lewy body dementia (LBD). Additionally, one of the studies investigates hypopituitarism (HPs), a condition linked to an increased risk of developing non-alcoholic fatty liver disease (NAFLD) and its more severe form, non-alcoholic steatohepatitis (NASH), due to growth hormone deficiency (GHD). Another study in this Research Topic seeks to assess the causal relationship between circulating lipids and the risk of urinary stones while examining also the role of lipid-lowering drugs, including HMG-CoA reductase inhibitors (HMGCR) and proprotein convertase subtilisin/kexin type 9 (PCSK9) inhibitors, in modulating this risk. Collectively, these studies underscore the value of lipidomic and multi-omics approaches in uncovering novel insights into complex diseases and their associated risks.

In the first article, Tan et al. investigated the causal relationship between plasma lipid composition and the occurrence of urinary stones, a major public health problem with a prevalence of 11% in certain populations and nearly 20% in the 60-69 age group. The available resource of genome-wide association study (GWAS) data from the UK Biobank initiative including 462,933 patients was used as a public cohort (1) to understand the association between plasma lipid set, generic variations and urinary stone occurrence by using Mendelian randomization (MR) statistics. The study revealed that an increased incidence of kidney stones was associated with rising blood triglyceride levels, while did not reveal any significant correlation with the levels of low-density and high-density lipoprotein cholesterol, lipoproteins (i.e., apolipoprotein A, apolipoprotein B) and lipid-lowering drugs.

The alteration of plasma triglyceride levels was also found to be relevant by Huang et al. for the stratification of metabolic syndrome (MetS) and the pre-metabolic state (Pre-MetS) when using different machine learning algorithms (10). In particular, they identified two panels of metabolite biomarkers: PS(38:3), DG(16:0/18:1), which were significantly different between Normal and Pre-MetS, and TG(16:0/14:1/22:6), TG(16:0/18:2/20:4), TG(14:0/18:2/18:3), which could discriminate Pre-MetS from MetS.

In the third article Zhang et al. explored a multi-omics approach to studying non-alcoholic fatty liver disease (NAFLD) induced by growth hormone (GH) deficiency in individuals with hypopituitarism (HP). HP is a rare disorder characterized by the deficiency of one or more hormones produced by the pituitary gland. First, the authors performed untargeted metabolomics analysis on serum of 134 patients with HP and randomly selected controls, identifying differential metabolites associated with mitochondrial function and oxidative stress. In the second part of the study, they investigated hypophysectomized rat models of human HP, analyzing changes in liver tissue using label-free quantitative proteomics and phosphoproteomics. Functional enrichment and protein-protein interaction analysis of the differential liver proteome and phosphoproteome, along with additional biochemical experiments, revealed dysregulation of the Jak2-Stat5B and mTOR pathways, accompanied by increased oxidative stress and lipid peroxidation in the rat livers. Consistent with the elevated oxidative stress markers observed in the serum of individuals with hypopituitarism, the authors conclude that oxidative stress and lipid peroxidation contribute to the development of hepatic steatosis and steatohepatitis in GH deficiency-induced NAFLD.

While the first two studies emphasized the role of altered triglyceride levels in distinguishing disease states, the final paper by Fu et al. shifts the focus to the pivotal role of lipoproteins in disease development. The authors investigated Lewy body dementia (LBD) (11, 12), a neurodegenerative disorder characterized by the accumulation of Lewy bodies, abnormal protein aggregates primarily composed of misfolded alpha-synuclein (αS), and linked to lipid metabolism dysfunction, particularly in carriers of the APOE4 allele. The findings reveal that both low-density lipoprotein cholesterol and remnant cholesterol (RC) significantly increase LBD risk, with RC posing a higher risk in APOE4 carriers. Human plasma lipidome analysis (13) revealed specific phosphatidylcholine (PC) and phosphatidylinositol (PI) components as key factors, with PC (O-16:0_20:4) and PC (O-18:1_20:4) showing protective effects against LBD, while PI (18:1_20:4) was associated with increased risk.

This Research Topic focuses on cutting-edge omics approaches for the study of lipid-rich human samples. By enabling comprehensive and precise molecular characterization, these methods hold the potential to drive transformative metabolic discoveries with far-reaching implications for society and healthcare.
